# Human milk products in the National Health Service: a cross-sectional survey of use and industry contact across England's trusts

**DOI:** 10.1177/20542704241237658

**Published:** 2024-04-30

**Authors:** Sarah L. Steele, Noah C.A. Cooke

**Affiliations:** 1 Health and Social Care, 2591 University of Essex, Colchester, UK; 2 Cambridge Public Health, Forvie Site, 98947 University of Cambridge, Cambridge School of Clinical Medicine, Cambridge, UK; 3 St Edmund's College, Cambridge, UK; 4 Department of Social and Political Sciences, Bocconi University, Milan, Italy; 5 Hughes Hall, 2152 University of Cambridge, Cambridge, UK

**Keywords:** Human milk, NHS, donor milk banks, regulation, first food systems

## Abstract

**Objectives:**

Commentators and professional organisations note that an expanding market in human milk-based products (HMBPs) could reduce breastfeeding, compromising maternal and infant health, and undermine public milk bank donations. We investigate whether English NHS trusts purchased these products and whether HMBP companies have marketed to them.

**Design:**

Freedom of Information (FOI) requests asking: (1) whether trusts obtained human milk; (2) if so, how; and (3) whether HMBP companies had approached them. We analysed trusts’ responses qualitatively. In 2023, an FOI request to the Food Standards Authority (FSA) following a product recall.

**Setting:**

England.

**Participants:**

One hundred and ninety-four NHS trusts, the FSA.

**Main Outcome Measures:**

Obtaining human milk, approaches by companies, and trust responses to approaches.

**Results:**

One hundred and seventy-six trusts responded, 102 reporting human milk from milk banks. No trusts reported purchasing from companies in 2022. In 2023, the FSA confirmed six English hospitals used HMBPs from one company; an FOI for trusts’ names was refused on law enforcement grounds. Two trusts reported participating in clinical trials funded by companies. Twenty-one reported approaches, using several strategies, including uninvited ward visits. Trusts rejected marketing based on guidance from: (1) trust dieticians or physicians; (2) regional regulatory bodies; (3) professional bodies; and (4) perceived application of an International Code on breastfeeding.

**Conclusions:**

Companies market to trusts, adopting methods previously used by the formula industry. Trusts express confusion over whether this infringes agreements designed to promote breastfeeding. We encourage clarification and guidance for professionals and trusts to ensure safety, infant and maternal health, and protect public provision.

## Introduction

Public health researchers, professional bodies and non-governmental organisations (NGOs) have expressed concern over commercialising human milk.^1–4^ Human milk-based products (HMBP) are now produced and marketed by private companies operating in several countries, including the USA,^5–9^ the UK,^10–12^ Brazil,^13^ Australia and India.^14^ Ready-to-drink and powdered human milk, fortifiers and synthetic products derived from human milk are marketed. Increasingly, the sale of these products has become an issue for national governments and international bodies, following a high-profile ban in India.^15^

Such controversy emerged because these HMBPs often require large volumes of human milk. Cambodia banned the export of milk in 2017, after reports gained the attention of the international press and UNICEF, when women from socioeconomically deprived backgrounds were being paid to pump milk for export and processing in the USA, where it was being sold at high prices to caregivers and hospitals.^16^ In July 2022, India went further, stating milk should *only* be altruistically given, and later in the year, the High Court ordered a writ removing the licence of its only HMBP company following acknowledgement it had been paying non-government organisations to recruit women in rural deprived areas to ‘donate’ their milk.^15^ UNICEF, various researchers, and commentators, have therefore questioned the ethics of HBMPs and their impacts on infant and maternal health.^16,17^

In England, the marketing of HMBP products led the Baby Feeding Law Group UK, an association of 30 members, including health professionals and NGOs, to express concern that HMBP could undermine NHS provision, if companies attract mothers to sell milk rather than donating it to the NHS or non-profit milk banks, while also potentially displacing breastfeeding.^2,18^ The International Code of Marketing of Breast Milk Substitutes (the Code) impresses upon countries the need to protect and promote breastfeeding, ‘ensuring the proper use of breastmilk substitutes, when these are necessary’,^19^ but nevertheless studies in Europe identify numerous gaps or lack of clarity around HMBPs’ legal status and regulation, leading for calls for law reform.^20,21^

Considering such concerns and calls, we investigate the uptake of these products in the English NHS and whether HMBP companies are approaching and marketing to NHS trusts and professionals. We also ask: How are NHS trusts responding? To answer these questions, we undertook a cross-sectional survey of NHS trusts using Freedom of Information (FOI) requests and also, following extensive press in the UK about a product recall by the Food Standards Authority (FSA), a follow-up FOI request to that agency for data on hospital usage.

## Methods

In December 2021, we emailed requests to NHS trusts in England, identified using the NHS website,^22^ with the NHS selected as it is one of the world's largest public healthcare systems and is subject to the Freedom of Information Act 2000 (FOI Act).^23^ This Act allows individuals to request data held on spending, policy and practice, as well as purchasing and approaches by industry.^23^ FOIs enable the systematic generation of data about public service practices, facilitating transparency and good governance. Notably, upon receipt, the public body has 20 working days to respond either by providing the requested data or applying an exemption per the Act, which includes, amongst other exemptions, that the information was previously requested via FOI and accessible via the publication scheme, answering the request is too costly or labour intensive (exceeding £450 or 18 staff hours for the NHS), or that the disclosure of information is against public interest or likely to result in prejudice.^23^ As such, because there is a legal obligation to respond to an FOI,^23^ response rates tend to be high and achieved within a short timeframe.

We downloaded a list of trusts from the NHS website and excluded trusts that were clearly ambulance or mental health trusts, thereby excluding those obviously not involved in infant feeding. We also note that during our study some trusts combined into a single hospital trust (e.g. reducing the overall number of trusts included in our study), while other trusts had incorrect email addresses on websites or had inactive website submission forms, precluding their inclusion into the study. We therefore successfully contacted a total of 194 English NHS trusts with an FOI request.

Our requests asked the trusts: (1) whether the trust acquires human milk for infant or paediatric feeding, and if so how; (2) whether the trust acquires products from private companies, and if so, how much was spent across the past five years and (3) if the trust did not currently use private suppliers, if any companies had approached it about HMBP. Where responses did not follow the FOI structure or were ambiguous, to reduce bias, we sought clarification from the relevant information governance officer or entered data in line with the original FOI question structure where possible. Notably, we sent a further FOI to the FSA in March 2023 following news reports that one HMBP company in England was subject to a product recall requesting any data they held on hospitals using these products.

Responses were collated into an Excel sheet for analysis of how many trusts purchased milk and to enable qualitative exploration of any industry approaches to healthcare providers. When reporting qualitatively, we identify the trust by region rather than name and removed any names of individuals to protect confidentiality.

## Results

A total of 175 NHS trusts responded to the FOI by October 2022, with 30 indicating that the FOI was irrelevant to their trust (e.g. because they are a specialist trust) or did not hold any of the information. Of the 175, 102 trusts identified using human milk in their hospital, and all 102 used NHS or charitable milk banks to obtain human milk for infant feeding. No NHS trusts reported using private companies to obtain human milk in 2022. One trust reported currently being involved in clinical trials with a fortifier product produced by a UK company, NeoKare Nutrition Limited, which was funding the trial. Another trust described participating previously in a clinical trial funded by a US-based company, Prolacta Bioscience.

**Figure 1.**
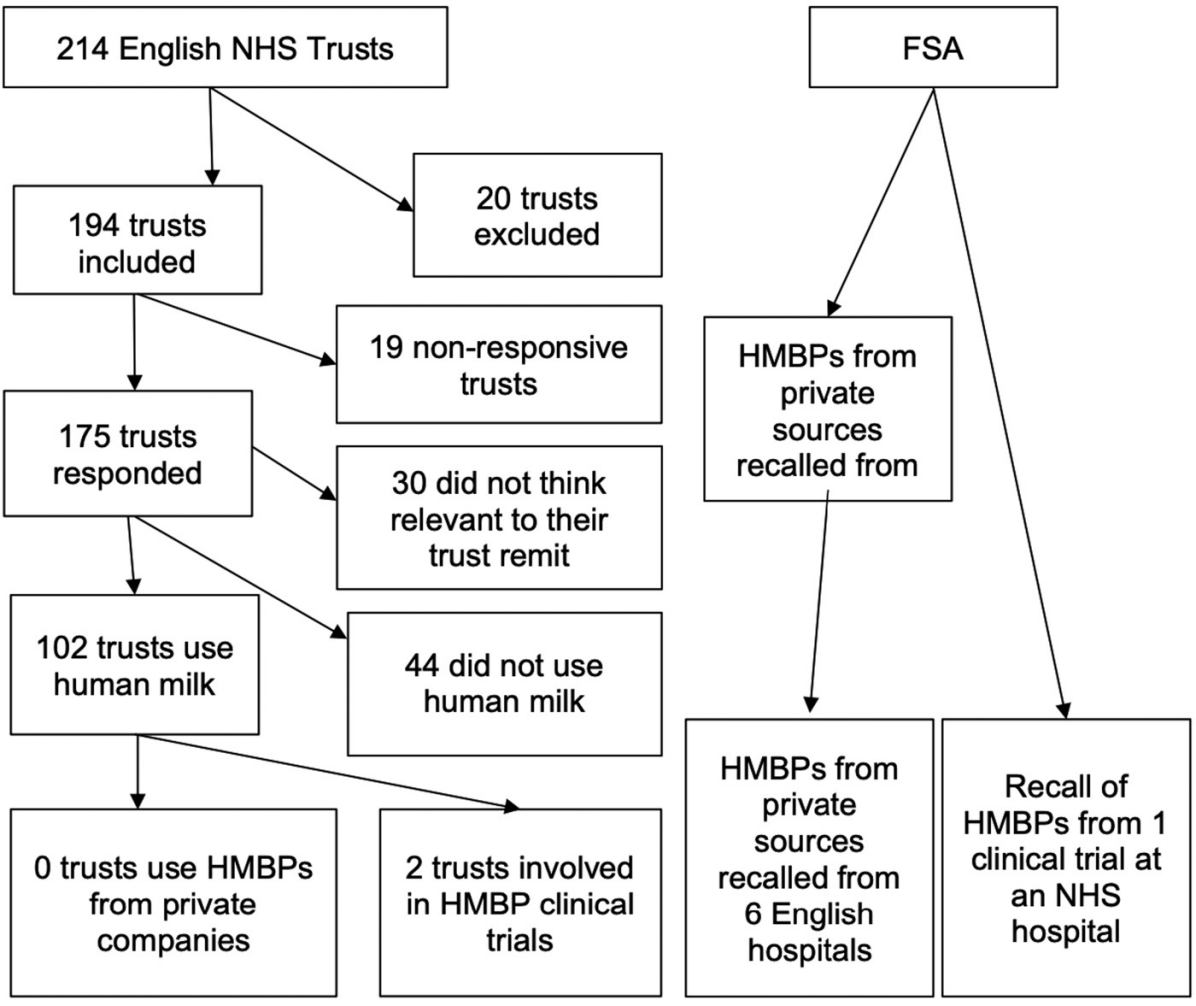
Freedom of Information requests sent and responses received.

The FSA refused our request for trust names because law enforcement action was pending, an exemption in the FOI Act, but provided a link to a report which provided the total number of hospitals in UK using the company's products in 2023, including specific numbers in England.^24^ The FSA reported that 13 hospitals had used the company's products at the time of the recall in 2023, including six English hospitals, with one trust engaged in a clinical trial (see Figure 1).

Our analysis also revealed that, when asked about being approached by a company or companies about HMBP, 21 trusts responded that they, or healthcare professionals in their trust, had been approached in some manner. Some trusts identified which companies had approached them: 13 identified NeoKare, five Prolacta, and two BestMilk. Six trusts did not specify the vendor. Notably, one trust replied that it had been approached by private companies but clarified that this was a non-NHS milk bank charity, so the researchers coded this as a negative response.

Qualitative analysis revealed the promotional activities of private companies targeted a range of entities within trusts. These included the trust itself, milk banks within trusts, Neonatal Intensive Care Unit (NICU) and/or Special Care Baby Unit (SCBU) teams, dietetic teams, and individual staff. For instance, a trust in the east of England described how their:

NICU team has not been approached directly about their products; however, some members of staff in the Paediatric Dietitian team have been approached individually.

Meanwhile, a trust in London disclosed that:

The Dietetic team have been approached by NeoKare.

As for what products were being promoted, a trust in north-west England disclosed that:

Neokare have approached members of the NICU team on several occasions promoting their human milk fortifier and 70 kcal milk.

Moreover, several modes of outreach were reported. For instance, a trust in the midlands noted that:

The human milk bank has been contacted by email on approximately two occasions in the last year from companies selling human milk.

Meanwhile, a trust in the south-west of England described a more aggressive approach on hospital grounds:

Neokare have attempted to come onto the Neonatal unit to speak staff, however they were sent away as we do not see reps from formula companies without appointment (as per BFI/Unicef code).

Here, the attempt was rebuffed because the trust perceived human milk products to be subject to the same international agreements as formula.

Other trusts activated policies from various levels of authority when responding to private companies. A different trust in the east of England explained that:

SCBU and NICU Ward Managers were approached by a private company in 2020. We did not purchase EBM [Expressed Breast Milk] from this company. The East of England Operational Delivery Network advised the neonatal units in the region not to engage with company.

This suggests that a regional policy exists on engagement with HMBP companies. Meanwhile, another trust in the Midlands noted a policy at the level of a neonatal network, of which there are 13 in the UK.^25^ The trust stated:

We have been approached by NeoKare. The guidance from our neonatal network is to direct any inquiries to the neonatal network dietician [Name] which is what we did in this case.

Similarly, a different London trust reported:

Neonatal units around London have been approached by NeoKare. LNODN [London Neonatal Operational Delivery Network] have warned all units not to use this company.

The trust provided a document from the British Dietetic Association (BDA) and its Neonatal Dieticians Group.

The BDA document attached to the response noted several issues of concern around marketing, safety and efficacy, a lack of adherence to national guidance, and cost, while noting:

reports from neonatal units of unsolicited contact by the company and their representatives attempting to promote their products.

It then made several cautions relating to safety and efficacy:

NeoKare are yet to release any published UK data on safety, tolerance and efficacy in preterm nutritional management in respect of Neokare MMF [Mother'sMilk Fortifier] or other human milk-based products available from Neokare.

Most concerningly:

Milk providers are screened as per NICE Guidance, but the products are manufactured from pooled human milk samples and as such are not compliant with the full 2010 NICE donor milk guidelines.

Further concerns were raised that:

NeoKare costs £22.50 per sachet (25 mL EBM [Expressed Breast Milk), that's £135 per day for a 1 kg infant receiving 150 mL/kg/day MEBM [Mother's Expressed Breast Milk], 100 times more expensive than bovine fortifiers.

However, the level of influence this document had on the trust's decision was unclear, as the response focused on LNODN advice.

For other trusts, it was unclear in their response whether a policy was followed. Another trust in the southwest of England described deliberation between the dietician and the relevant medical team in responding to approaches:

A representative for breast milk fortifier made from breast milk has in the past via email/phone conversation approached [trust]. Following a discussion with the dietician and infant feeding Team it was decided the product available on the market is not suitable for [trust] use.

Two trusts mentioned that dieticians were consulted in their responses, suggesting an intention to ascertain whether the products could benefit their patients. In both cases, the dieticians advised against purchasing the products.

Other answers from trusts suggest that satisfaction with current milk bank supplies led to declining offers from private companies. For instance, one trust in the south-east of England reported that its hospitals:

have been approached by NeoKare but currently happy with supply from [another NHS hospital] …Happy with current arrangements.

## Discussion

### Principal findings

Our results show that the NHS continues to rely on donor milk banks to provide human milk for neonates across England who need it. However, according to the FSA, fortifier products made by one HMBP were recalled from usage in six English hospitals in 2023.^24^ HMBP companies are actively marketing to NHS trusts and their employees. The reasons why trusts denied these approaches vary. Some were satisfied with existing providers; others acted on the advice of neonatologists and dieticians, professional bodies, or local authorities; and other trusts perceived marketing attempts as contrary to international regulations on breastmilk substitutes.

The invoking of the Baby Friendly Initiative (BFI) and the Code by two trusts, despite a lack of clarity around its application to HMBPs made from human milk, underscores a more significant issue: insufficient regulatory guidance. The Code is, in fact, silent on HMBP, partly because it was devised in 1981 before the first HMBP came to market. It is unclear whether the Code, which aims to protect breastfeeding, could be interpreted as placing some or all of these products within its definition of infant formulas and foods, or as breastmilk, which it seeks to promote as optimal infant nutrition.^26^ If HMBP were included within the Code's purview – and so considered as products that may displace breastfeeding – the promotion of any product in a healthcare facility, direct approaches to healthcare providers on the ward, and direct marketing to caregivers, would all be prohibited.^19,26^ Critically, letters to UK regulators have suggested that HMBP products may displace breastfeeding itself, an outcome contrary to the spirit and purpose of the Code, and professional organisations like the BDA appear to support this view. Research is needed on whether some or all HMBP indeed displace breastfeeding and under what circumstances, as well as legal research on interpreting the Code.

### Strengths and weaknesses

Our cross-sectional FOI survey enabled us to detect and characterise direct marketing approaches by HMBP companies to the largest public healthcare system globally.^27^ Our study, though, is subject to several limitations. The difference between our results and the FSA's recall number may be due to difference in counting (trusts versus hospitals) or may indicate under-reporting by trusts, either by mistake, because of free provision of HMBP (e.g. as samples), usage being in reported clinical trials, or potentially the success of the aggressive marketing techniques in expanding purchasing by 2023. Our follow-up FOI to the FSA was refused due to pending law enforcement action, preventing confirmation of: (1) trusts purchasing or accepting HMBP as samples or in trials and (2) trusts’ expenditure on recalled products. Future research may further illuminate NHS market intrusion alongside further marketing activities. Second, our analysis cannot be comprehensive: it draws on FOI requests, rather than documents discovered through purchase orders or court proceedings, which prior studies suggest may be more comprehensive.^27^ Third, our findings pertain mainly to hospital-based NHS trusts and cannot be generalised to other primary care and community-based healthcare services. Future research is needed to investigate approaches to general practices, health visitors (e.g. specialist community public health nurses), midwives, and lactation consultants.

### Implications and future directions for research

The attention of national and international regulators is critical considering India's ban, mainly because the company whose licence was revoked was the Indian sister company of a UK-registered company.^28^ International relations issues could arise where one country actively supports the set-up and growth of a company in its jurisdiction when that company is de-licenced in another because of human rights and health concerns. International organisations may seek to expand the Code to include some or all HMBP. Alternatively, international organisations may separately seek to address HMBP, addressing its unique human rights facets. Either way, clarity must be reached on whether HMBP are within the Code's remit.

At a national level, regulators and policymakers must address HMBP, and should consider UNICEF's statements supporting Cambodia's export ban, and the multiple human rights, health and development impacts it identified.^16^ In England, many trusts described decentralised decision making in their responses: by local health professionals, or guidance from local or regional professional organisations like the BDA. Trusts’ responses suggest that a lack of HMBP-specific guidance from NICE is leaving trusts without a national framework of evidence-based policy to guide their decisions on both HMBP themselves and responses to marketing. NICE could appropriately weigh the benefits and risks of HMBP – both clinical and ethical – and issue guidance to standardise policies across trusts. It is beyond our scope to assess whether any HMBP – indeed, any HMBP marketed to trusts in this study, or under study in clinical trials – would benefit infants, or subgroups like low-weight pre-term infants. However, as national guidance is not yet in place, we alert healthcare professionals to HMBP marketing, about which several professional bodies, including the British Dietetics Association, have raised concern.

The marketing of breastfeeding substitutes also needs to be reformed. Research and international organisations readily acknowledge that the companies in the infant feeding space have gone to great lengths to ‘secure the recommendation’ of healthcare professionals and providers.^21^ Also, to enable its marketing approach, the global formula industry has engaged in practices such as lobbying international and national policymakers, to foster favourable regulatory environments.^21,29^ The targeting of healthcare professionals, rather than NHS Supply Chain – the body that manages the sourcing, delivery and supply of healthcare items and food for the NHS – therefore suggests that HMBP companies may be employing strategies well-known of the formula industry. We encourage researchers to explore further marketing, industry influence and conflicts of interest in the human milk space.

Policymakers must also consider how trusts should plan for and respond to milk bank shortages in the short-term. Several trusts, satisfied with current providers, rebuffed marketing approaches, but this may change. Demand for human milk grew over the pandemic, straining milk banks supplies.^30^ Moreover, public supply could diminish if for-profit HMBP companies displace donations. Under such circumstances, we wonder whether trusts would respond positively to industry approaches. Furthermore, policymakers should consider regulating direct marketing to caregivers. Future studies should explore NHS and caregiver usage following the recent product recall and the influence of claims about NHS usage on caregiver decisions to use HMBPs for infant feeding.

## Conclusion

The responses of NHS trusts to our FOI requests suggest that HMBP companies did not sell to the NHS before October 2022, while the FSA results suggest only six English hospitals have used one company's products, not making clear whether these products were purchased or otherwise acquired. However, HMBP companies have approached at least 21 trusts, using several marketing strategies. Notably, two trusts have engaged in clinical trials, one affected by the product recall.

Our study contributes a vital understanding that, in the largest public healthcare system worldwide, HMBP companies appear to have employed certain marketing behaviours reminiscent of those companies in the wider infant feeding and pharmaceutical spaces, which have been deemed problematic.^29^ Our study has important implications for the NHS, healthcare systems worldwide, and national and international regulatory bodies. Existing regulations and guidelines need to be revised. In the short-term, policies could be developed to guide trusts in responding to potential shortages in public supply. In the long-term, guidelines and regulations, based upon an evidence-based evaluation of HMBP, should be considered to best serve infant and maternal health, protect the public provision of milk, and uphold human rights. Options include revising the Code to include some or all HMBP, developing separate regulations for HMBP, and developing regulations to limit direct marketing of HMBP to caregivers, healthcare professionals or individual NHS trusts.
